# On the use of data mining for estimating carbon storage in the trees

**DOI:** 10.1186/1750-0680-8-6

**Published:** 2013-06-10

**Authors:** Carlos Roberto Sanquetta, Jaime Wojciechowski, Ana Paula Dalla Corte, Aurélio Lourenço Rodrigues, Greyce Charllyne Benedet Maas

**Affiliations:** 1 Department of Forest Science, Federal University of Paraná, Rua Simão Brante, 103, sob. 5, Uberaba, Curitiba, Paraná, 81.570-340, Brazil; 2 Sector of Professional and Technological Education, Federal University of Paraná, Curitiba, PR, Brazil; 3 Graduate Programme in Forestry, Federal University of Paraná, Curitiba, PR, Brazil

**Keywords:** Biomass, Biomass expansion factor, Euclidean distance, Regression equations, Root-to-shoot ratio

## Abstract

Forests contribute to climate change mitigation by storing carbon in tree biomass. The amount of carbon stored in this carbon pool is estimated by using either allometric equations or biomass expansion factors. Both of the methods provide estimate of the carbon stock based on the biometric parameters of a model tree. This study calls attention to the potential advantages of the data mining technique known as instance-based classification, which is not used currently for this purpose. The analysis of the data on the carbon storage in 30 trees of Brazilian pine (*Araucaria angustifolia*) shows that the instance-based classification provides as relevant estimates as the conventional methods do. The coefficient of correlation between the estimated and measured values of carbon storage in tree biomass does not vary significantly with the choice of the method. The use of some other measures of method performance leads to the same result. In contrast to the convention methods the instance-based classification does not presume any specific form of the function relating carbon storage to the biometric parameters of the tree. Since the best form of such function is difficult to find, the instance-based classification could outperform the conventional methods in some cases, or simply get rid of the questions about the choice of the allometric equations.

## Background

Forests play an important role in the global carbon balance, fixing carbon from the atmosphere, but can also become a source of emissions [1-3]. Therefore, their function is critical to global efforts to combat climate change. Quantifying carbon storage in forest ecosystems and the resulting changes stemming from human activities is a first step to better represent forests in climate policy at regional, national and global scales [4].

The estimates of biomass and carbon in forests can be made by direct and indirect methods [5-9]. Direct methods consist of weighing all biomass, which is typically a destructive process. This approach is not feasible for large-scale applications, due to time and implementation cost constraints. Meanwhile traditional indirect methods imply the use of some type of biomass and carbon modeling, using expansion factors and/or regression equations. However, such factors or equations need to be fitted using primary empirical data.

Regression equations whose dependent variable is individual tree carbon *(C)* and whose independent variables are tree measurement parameters, are often called allometric equations [10]. In most instances they are appropriate because they offer direct estimates of *C* as functions of tree measurements, which are easy to measure and apply. However, they are not always satisfactory in terms of accuracy, and result in error levels beyond tolerance thresholds in forestry measurements.

The other traditional method consists of fitting bole volume *(V)* equations, which is commonly estimated in conventional forest inventories and usually obtained with high precision and accuracy, combined with factors that extrapolate the bole mass to estimate the entire aboveground as well as below ground biomass. These factors are called the Biomass Expansion Factor *(BEF)* and the Root-to-Shoot Ratio *(R)*[11].

According to Soares & Tomé [10], allometric equations are recommended when estimating forest carbon, but if not available we can make use of expansion factors specific to different ages of forest stands. The limitations of biomass expansion factors are discussed by the authors.

Data mining aims to discover useful information in a data set [12]. This technique, used in learning algorithms whose metrics can be found in [13] and Bradzil et al. [14], is now widespread in many areas and applications. However, its potential has not been exploited to estimate carbon stocks in forests. We decided to evaluate this technique because it allows one to better explore the nature of the numerical data, is simpler and more flexible than classical regression modeling because it did not require testing preconceived mathematical formulae, and also because it may yield estimates of individual tree carbon as accurate as those obtained from classical allometry.

This study aims to evaluate the technique of data mining against the two most common procedures for estimating carbon in forests. The species *A. angustifolia,* Brazil’s most important native conifer, was used as a case study.

## Results and discussion

### Descriptive statistics and correlation matrix

Table 1 shows the descriptive statistics of the data used in this study. For trees aged 14 to 31 years, their diameters ranged from 14.29 to 33.39 cm and their height ranged from 12.72 to 19.90 m. The carbon stock of individuals ranged from 25.91 to 228.43 kg. The *BEF* ranged from 1.05 to 2.06, indicating some trees have low canopy biomass while others have much more since this fraction is larger than that of the bole itself. *R* in turn ranged from 0.03 to 0.12, indicating that the expression of underground biomass also varies among individuals.

**Table 1:** **Descriptive statistics for the variables analyzed for *A. angustifolia***

**Statistics**	***DBH *(cm)**	***H *(m)**	**Age (years)**	***BEF***	***R***	***C *(kg)**
Average	24.69	16.69	21.67	1.39	0.07	113.01
Minimum	14.29	12.72	14.00	1.05	0.03	25.91
Maximum	33.39	19.90	31.00	2.06	0.12	228.43
Standard Deviation	5.62	2.07	5.85	0.25	0.02	58.20
Coefficient of Variation	22.76	12.39	27.00	17.94	31.15	51.50
N	30	30	30	30	30	30

Silveira [15] points out that studies on the use of *BEF* to estimate forest carbon are scarce in Brazil. The author has worked with a wide range of native species in dense rain forest in the state of Santa Catarina, Brazil. Watzlawick [16], Sanquetta *et al*. [17] and Schumacher *et al.*[18] studied *A. angustifolia* biomass and carbon storage without evaluating *BEF* and *R.* In inferring biomass values presented by the authors, mean *BEF* and *R* values were 1.34 and 0.18 for stands aged 24–32 years in the the municipality of *General Carneiro*, Brazil [16]. Mean *BEF* and *R* values of 1.32 and 0.15, respectively, were found for a 27-year-old stand that had been clearcut in the *Quedas do Iguaçu* municipality [18] and 1.39 for *BEF* in *General Carneiro*, in forest plantations with ages ranging from 14 to 32 years [17]. In *Pinus* stands in the state of *Paraná*, Sanquetta *et al.*[11] found average *R* and *BEF* values of 1.43 and 0.17, respectively, ranging from 0.05 to 0.63 for *R* and from 1.09 to 3.74 for *BEF*. Studies investigating biomass proportions in other species include that of Schneider and Finger [19] in *Acacia mearnsii* and Miranda *et al.*[20] in *Euterpe oleracea*.

The correlation matrix between variables (Table 2) showed that the total individual carbon stock (*C*) is more strongly correlated to *DBH*, and then to tree height and age. These correlations were positive and statistically significant (*p*<0.05). This means that the larger and older the tree, the greater its carbon stock, which is to be expected. The correlation between *C* and *BEF* was negative but the correlation between *C* and *R* was positive, though neither were statistically significant (*p*<0.05). This means that carbon stocks of the individual were not affected by the canopy proportions (branches + foliage) nor underground biomass (roots) in this case. *BEF* and *R* did not share significant correlations. *BEF* was negatively correlated with age and *R,* and positively correlated with *DBH*. That is, with increasing age the proportion of canopy biomass tends to decrease, and as the tree grows with respect to *DBH*, so does the relative proportion of the roots. The data presented by Watzlawick [16] suggest similar behavior. Results for *Pinus taeda* in *Paraná* show a reduction in *BEF* and *R* with age and with *DBH*[11]. This possibly results from the fact that the minimum age considered in this study was 14 years.

**Table 2:** **Simple correlation matrix for the variables *DBH*, *H*, Age, *BEF*, *R *and *C *for *A. angustifolia***

**Variable**	***DBH***	***H***	**Age**	***BEF***	***R***	***C***
*DBH*	1					
*H*	0.86	1				
Age	0.57	0.63	1			
*BEF*	−0.36	−0.45	−0.67	1		
*R*	0.73	0.55	0.38	−0.44	1	
*C*	0.92	0.79	0.65	−0.26	0.54	1

Ratuchne [21] studied several species in Araucaria Forest and found correlation coefficients of total individual biomass with tree measurement variables similar to this study, but lower than those presented here. The reason is that the author analyzed a wide range of species.

#### Regression equations

The fitted carbon equations yielded coefficients of determination of between 0.85 and 0.86, and standard errors of the estimate near 20%. Among the models we tested the one resulting in the best fit was equation 1, though all four were satisfactory (Table 3). Schumacher *et al.*[18] obtained higher R^2^ values and lower Syx% values in modeling the total biomass of *A. angustifolia* individuals. This is likely due to the fact that the authors used data from a homogeneously aged stand, *e.g.* 27 years. Sanquetta *et al.*[17] found satisfactory fits for the biomass in the bole as a function of *DBH* and *H,* with low standard errors of the estimate and high coefficients of determination, though unsatisfactory in making partial biomass estimates in each of the compartments.

**Table 3:** **Statistical values of the fitted equations of total carbon for *A. angustifolia***

**Model**	**β**_**0**_	**β**_**1**_	**β**_**2**_	**R**^**2**^	**S**_**yx**_	**S**_**yx%**_
			Carbon			
1	−1.2538	2.3474	-	0.86	21.70	19.20
2	7.2153	0.0095	-	0.85	22.76	20.14
3	−1.7967	0.9506	-	0.85	22.80	20.18
4	−1.5232	2.1400	0.4559	0.86	22.32	19.75
			Volume			
1	−3.7737	2.4515	-	0.96	0.07	18.73
2	0.00025	0.00004	-	0.99	0.04	9.36
3	−3.8757	0.8665	-	0.94	0.09	22.51
4	−3.8483	1.9593	0.5979	0.95	0.08	21.01

**Figure 1:**
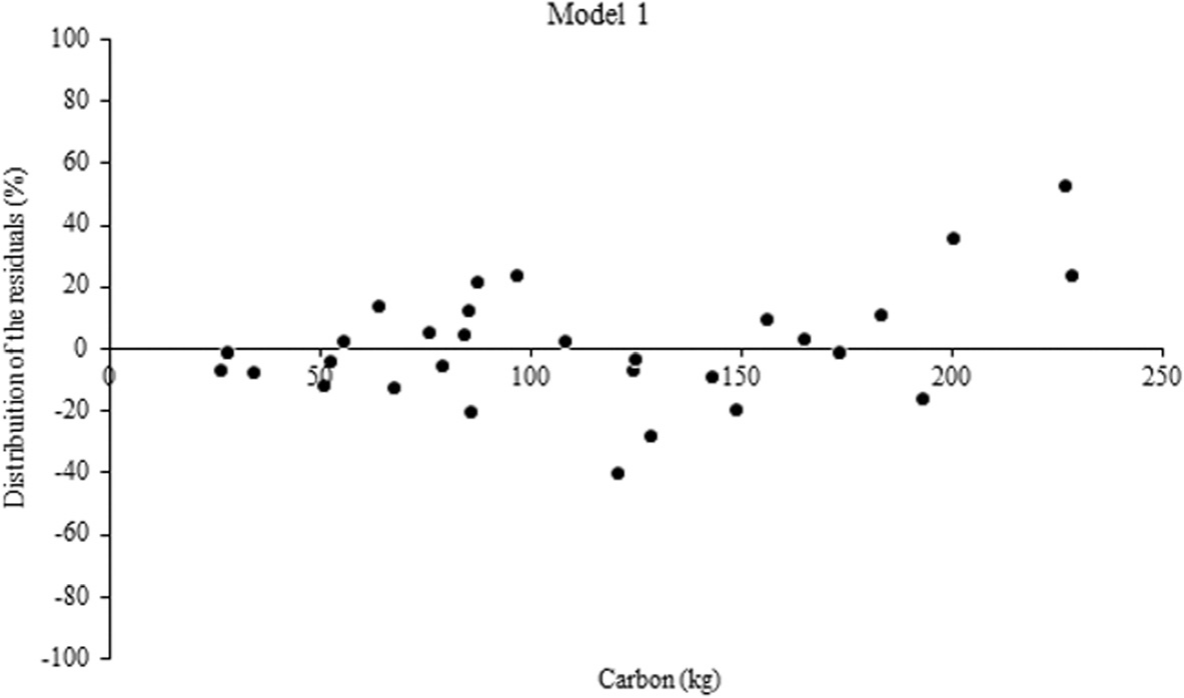
**Residual graphical analysis of the best performing carbon equation for *A. angustifolia.***

The volume equations yielded coefficients of determination from 0.94 to 0.99, with standard errors of the estimate ranging from 9 to 22%. The model with the highest R^2^ and lowest Syx% was Equation 2 (Table 3). The best performing residuals of carbon and volume models are shown in Figures 1 and 2. Several authors have conducted studies on fitting volume equations for the species we studied, such as De Oliveira et al. [22], De Mattos et al. [23], Schneider & Finger [19], among others. In most cases fits are satisfactory and comparable to this study, primarily due to the regular shape of the bole of the species.

**Figure Figure 2:**
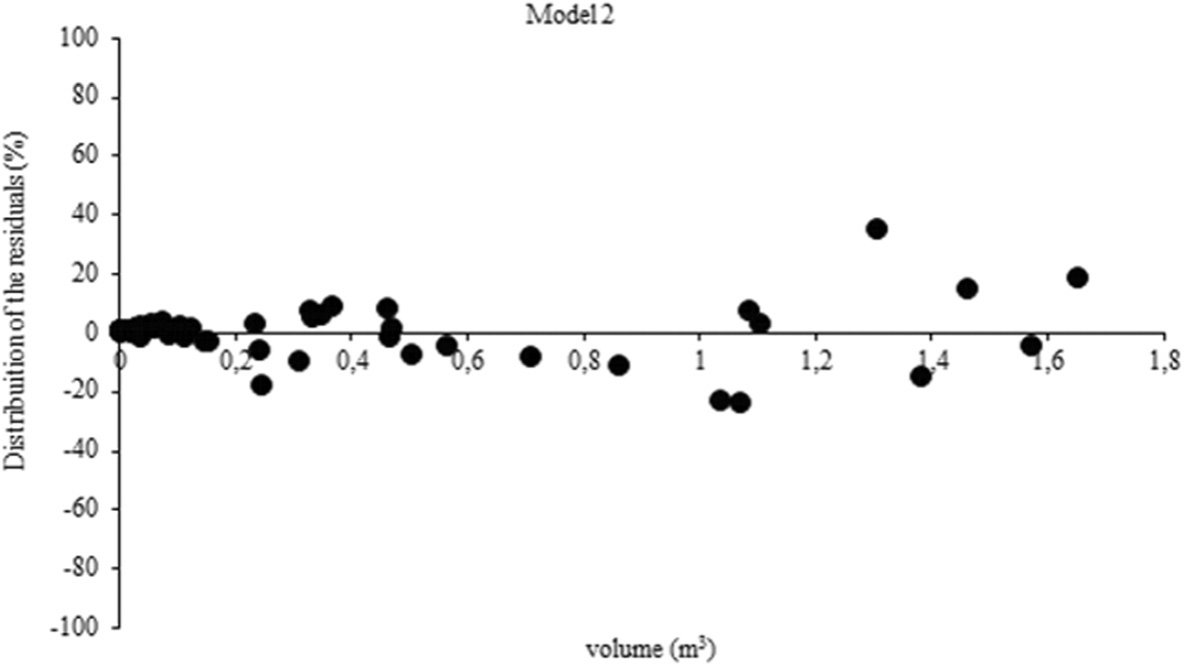
**Residual graphical analysis of the best performing volume equation for *A. angustifolia.***

#### Data mining

**Figure 3:**
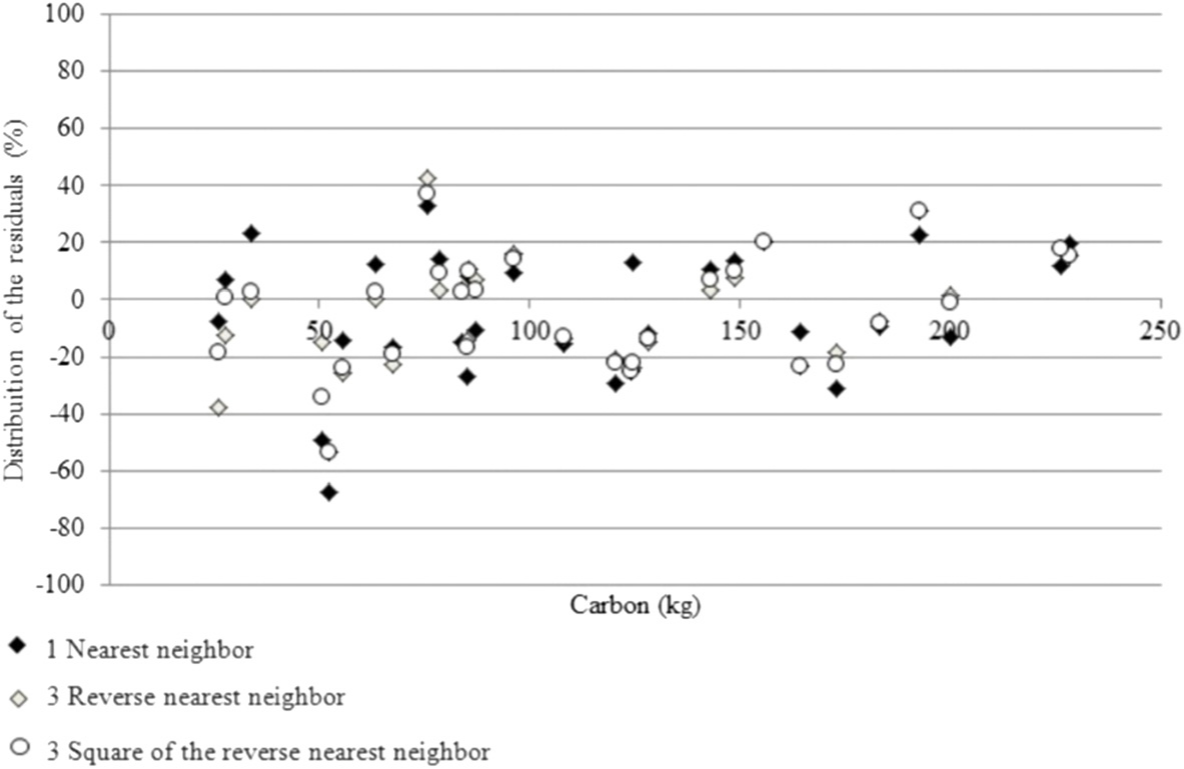
**Residual graphical analysis of three data mining methods for estimating carbon in *A. angustifolia *individuals.**

The three data mining methods analyzed in this study yielded similar results, with standard errors (Syx%) of 24.85% for the first procedure (one nearest neighbor), 24.10% for the 2nd procedure (inverse Euclidean distance weighted with 3 neighbors) and 24.32% for the 3rd procedure (inverse of the squared Euclidean distance weighted with 3 neighbors). All the R^2^ values were also similar, near 0.85. This suggests that any of the methods can be used satisfactorily. A graphical analysis of residuals revealed that there were no trends nor atypical dispersion in the residuals in any of the three methods (Figure 3).

**Figure 4:**
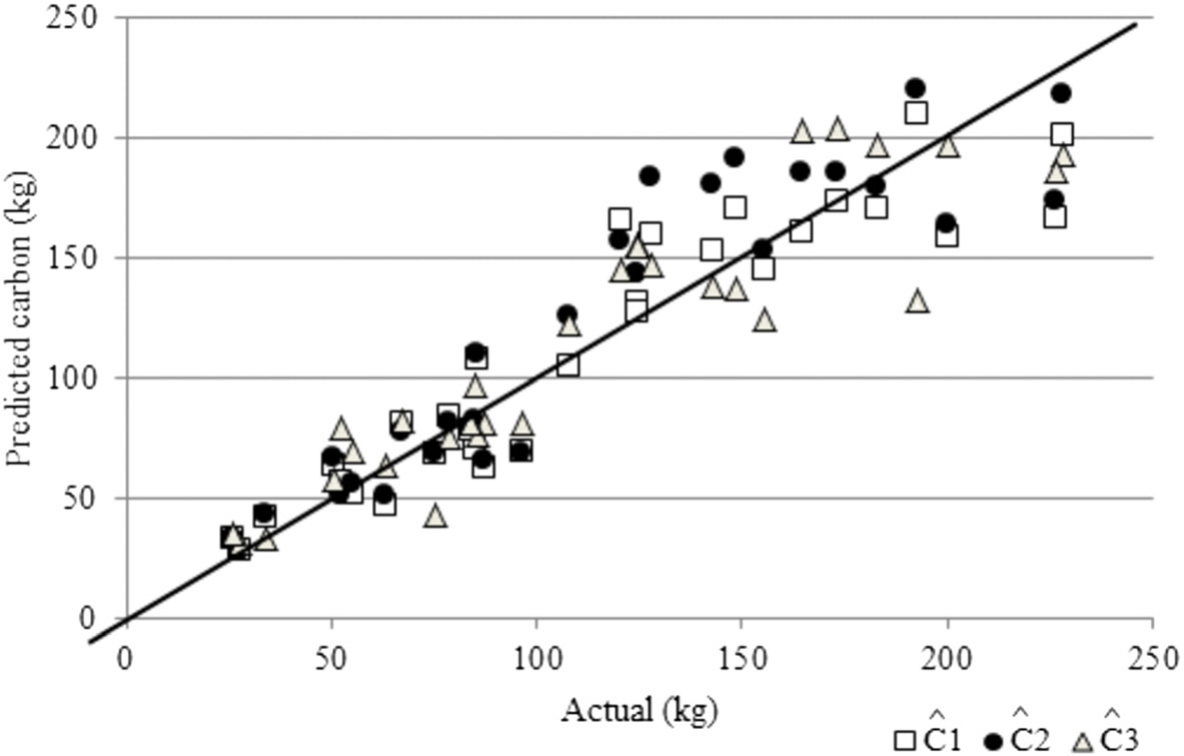
**Predicted vs. actual values of carbon stock of *A. angustifolia *individuals of the three methods tested (*Ĉ***^***1***^**: regression equation; *Ĉ***^***2***^**: volume equation and expansion factors; *Ĉ***^***3***^**: data mining).**

#### Comparative analyses

When the carbon stock estimates for individuals of the species were analyzed by using the best regression equation (*Ĉ*_*1*_) and the respective real values of this variable, we found that the average difference was −1.22%, *i.e.,* the average real value was 113.01 and the average estimated value was 111.63 kg (p = 0.7248). When comparing the estimates with Eq. 3 (*Ĉ*_*2*_), which were based on combining the estimated volume (in this case the second model was used as it performed better) and the expansion factors *(BEF* and *R)* and the respective real values of *C,* an average difference of 5.28% was found, with average estimated value of 118.98 kg (*p* = 0.0859). Comparing the alternative method of data mining (*Ĉ*_*3*_) resulted in a difference of 1.20%, with an estimated value of the variable of interest of 114.37 kg (*p* = 0.7556).

These differences, when analyzed by paired *t* test (*p* <0.05) were considered not significant, *i.e.,* both estimates (*Ĉ*_*1*,_*Ĉ*_*2*__and_*Ĉ*_*3*_) were not statistically different from the actual value of *C.* Figure 4 illustrates the ratio of the actual values of *C* and its estimates by traditional methods (regression of the carbon equation and the volume equation combined with volume expansion factors) and the alternative method (data mining). The simple Pearson correlation’s coefficients of actual *C* and the three estimates were 0.9308; 0.9232, and 0.91455, respectively for *Ĉ*_*1*_, *Ĉ*_*2*_, for *Ĉ*_*3*_.

Obtaining the forest carbon stock is not feasible using direct methods. Therefore it is important to consider the relationships between tree dimensions that are easily obtained, such as diameter, height and age. There are several ways to estimate the carbon stocks in trees. The most traditional uses allometric equations and the applicatin of expansion factors [10]. According to the authors, using allometric equations to estimate biomass is preferable, but when unavailable, expansion factors for different ages can be used. Sanquetta *et al.*[3] found a clear dependent relationship between both *BEF* and *R* and age, revealing negative curvilinear trends for *Pinus* in the state of Paraná. Similar results were obtained for *BEF* by Soares & Tomé [10] in *Eucalyptus globulus,* although its meaning is somewhat different (total aerial biomass divided by the volume of the bole).

Due to their simplicity and immediate application, both *BEF* and *R* have been employed alternatively to estimate biomass and hence carbon. They represent a simple way to extrapolate from the carbon in the bole, which is generally more easily obtained and estimated by multiplying the volume by the density of the timber, to the living biomass of the entire tree. Typically *BEF* and *R* are assumed to be constant, which is not always the case. The IPCC (Intergovernmental Panel on Climate Change) itself publishes tables with default medium *(default)* for various forests of the world IPCC - Intergovernmental Panel on Climate Change [24], since research on *BEF* and *R* is lacking for most of the world's forests, especially in the tropics. The use of *default* values (average) could pose problems for estimates of biomass and carbon, but often the only values available, since in many cases allometric equations are not available.

In the present study the estimates by the two traditional methods closely approximated each other. This occurred because a clear dependent relationship between *R* and *BEF* and age could not be observed nor indeed between the tree measurement variables *DBH* and *H*, as opposed to the previously mentioned studies (Figure 4).

The arguments of Soares & Tomé [10] are relevant when considering tree stands of varying tree ages. However it is important to note that the greatest variations in *BEF* and *R* occur at younger ages*,* due to the faster growth rates and the differences in allocation of biomass to different plant tissues as the plant matures. On the other hand, in intermediate and older stands that whose growth rates have stabilized and assume some archetypal form, *BEF* and *R* often remain constant with advancing age (as seen in the aforementioned studies). In this case the supposed advantage of allometric equations does not manifest as in the case of our study, which showed no statistical difference between the two methods for estimating the carbon stock in *A. angustifolia* individuals.

It is important to note that estimates of volume are always more accurate than estimates of carbon. Therefore, potential advantages of the methods may emerge from future discussion of this work. If the variable carbon does not depend on age and size of the trees *(DBH* and *H),* statistical gains are possible using the second carbon estimation method presented here, which is also relatively straightforward. The influence of the wood density variable in this context warrants further exploration.

An alternative method, which has rarely been explored in forest science, is data mining. The technique consists of an information management tool used to facilitate the access and organization of knowledge structures that assist decision-making. In practice it amounts to exploratory data analysis and modeling. According to Cardoso and Machado [25], it represents one of the most effective alternatives to extracting information from large volumes of data, revealing hidden relationships, patterns and generating rules to predict and correlate data that can help institutions make decisions faster and with a higher degree of certainty. The technique has been used in the health sciences [26] and business Marcanoo Aular & Talavera Pereira [27] industries, among others, with some forestry applications such as the prediction of fires [28] and mapping forest cover [29]. This technique allows one to better explore the nature of the data, is more flexible than the classical regression modeling because it requires testing preconceived mathematical formulations, and yields comparable and often more reliable estimates than classical allometry. Unfortunately there have been no studies applying the method to modeling forest carbon.

The results of this research indicate that the technique of data mining can be used to accurately estimate the carbon stock of *A. angustifolia* individuals*.* Its implementation is simple and yields estimates as satisfactory as those obtained by traditional methods of modeling carbon, such as equations fitted directly by linear regression or volume equations combined with expansion factors.

The use of data mining is shown as an initiative of the forest area to search techniques in other areas of knowledge that contribute in estimates of interest among researchers and foster the idea of diversifying methods in order to find better results, leaving the known techniques traditional and commonly used. Beyond data mining, other AI techniques have been studied with the same purpose. In Gorgens [30], Artificial Neural Networks were used to estimate tree volume and gave results compatible with traditional methods of fitting commonly used linear regression equations.

In other disciplines data mining has been used to classify dangerous parts of Brazilian cities, where useful information was found by employing decision trees for the attributes related to a particular characteristic such as infrastructure conditions, hydrology, soil, recreation area, community characteristics, among others. The goal of Malucelli et al. [31] was to classify parts of the city according to the degree of risk of residing in that area, indicating the potential for AI tools to be applied in any discipline including Forestry.

## Conclusions

In contrast to the convention methods the instance-based classification does not presume any specific form of the function relating carbon storage to the biometric parameters of the tree. Since the best form of such function is difficult to find, the instance-based classification could outperform the conventional methods in some cases, or simply get rid of the questions about the choice of the allometric equations.

## Methods

### Field data

The data used in this study come from pure stands of *Araucaria angustifolia* Bert. O. Ktze. located in the municipalities of *Foz do Iguaçu* and *General Carneiro*, southern Paraná, Brazil. The forest stand of Iguaçu Falls is located between the geographical coordinates 25° 20’ 58” a 25° 34’ 55” South and 52° 36’ 24” e 53° 07’ 43” West, and was installed between 1987 and 1988. Meanwhile the forest stand in *General Carneiro* is located between the geographical coordinates 26° 20’ 35” a 26° 26’ 13” South and 51° 19’ 49” a 51° 25’ 29” West, and was installed between 1969 and 1987.

Thirty trees were used for this study and were felled and weighed directly. Wet weights of all biomass were determined in the field in separate compartments: bole without bark, bark, branches, foliage and roots. Data from *DBH* (diameter at breast height) and total height *(H)* of trees were also collected. Samples of biomass of each compartment 300 to 500 g were collected for laboratory analysis. Samples were collected between the years 2003 and 2004.

#### Laboratory analysis

The fresh biomass samples were removed from the field and oven-dried to constant weight at a temperature of 70°C. Based on the relationship between wet weight and dry weight, the dry weights of all compartments of the biomass collected in the field were calculated. The samples were then chipped for analysis of carbon content *(T)* using the dry combustion method in an infrared chamber. Based on the carbon contents the carbon stocks in all compartments of the biomass were calculated, which together resulted in the total carbon *(C)* stock of the individual tree*,* the primary variable of this research.

### Expansion factors and wood density

From the field data Biomass Expansion Factors *(BEF)* and the Root Ratio *(R)* of trees were calculated using the following formulas IPCC - Intergovernmental Panel on Climate Change [32]:

(1) $$\mathrm{BEF}=\frac{P_{\mathrm{canopy}}+P_{\mathrm{bole}}}{P_{\mathrm{bole}}}=\frac{P_{\mathrm{aerial}}}{P_{\mathrm{bole}}}$$

(2) $$R=\frac{P_{\mathrm{roots}}}{P_{\mathrm{aerial}}}$$

Where:

*BEF* = biomass expansion factor (dimensionless);

*R* = root ratio (dimensionless);

*P*_*canopy*_ = dry weight of the tree canopy (kg);

*P*_*bole*_ = dry weight of the tree bole (kg);

*P*_*aerial*_ = dry weight of the tree bole + dry weight of the tree canopy (kg).

*P*_*roots*_ = dry weight of the tree roots (kg).

Conceptually, the *BEF* is a factor used to extrapolate the weight of the tree bole to the whole biomass, considering the branches and foliage. It is usually used to calculate the entire aboveground biomass using only volume (usually estimated in conventional forest inventories) and the wood density. Meanwhile the Root Ratio *(R)* is a factor that expresses the ratio of the below ground biomass to the above ground biomass, and enables one to calculate the weight of the whole tree's biomass.

For the purposes of this study an average wood density was fixed at 0.425 g.cm^-3^ De Mattos et al. [23]. This value, which expresses the relationship between weight and volume was used to calculate individual carbon stock based on bole volume and expansion factors *(BEF* and *R),* as will be discussed below.

#### Descriptive statistics and correlation analysis

We calculated descriptive statistics for the variables: *DBH, H*, Age (I), *BEF, R* and *C* and analyzed their correlations using a simple correlation matrix.

#### Methods for estimating carbon stock of individuals

Three different methods were used to estimate carbon *(C)* stock of the individual*.* The first two methods are traditionally used to that end, while an alternative method has been applied in other fields but never before been used to estimate forest carbon:

1. Estimation of Carbon Stock of Individuals from Regression Equations

In this approach *C* was estimated directly from a regression equation fitted to the field data, *i.e. C* was estimated directly as a function of *DBH* and *H* (see Eqs. 4, 5, 6 and 7).

Estimation of Carbon Stock of Individuals from Bole Volume and Expansion Factors

The carbon *(C)* stock estimates of individuals were calculated using the following mathematical expression, where *V* was estimated based on equations 4, 5, 6 and 7:

(3) $$C=V*D*\mathrm{BEF}*\left(1+R\right)*T$$

In both approaches the following regression models were tested:

(4) $$\mathrm{logY}=\beta_{0}+\beta_{1}*\log\;\mathrm{DBH}$$

(5) $$Y=\beta_{0}+\beta_{1}*\mathrm{DB}H^{2}*H$$

(6) $$\mathrm{logY}=\beta_{0}+\beta_{1}*\log\;\left(\mathrm{DB}H^{2}*H\right)$$

(7) $$\mathrm{logY}=\beta_{0}+\beta_{1}*\log\;\mathrm{DBH}+\beta_{2}*\log\;H$$

2. Where: Y = individual carbon stock *(C)* in method 1 or total bole volume *(V)* in method 2; *DBH* = diameter at breast height, *H* = total height; β_i_ = coefficients of the models.

One of the fitted equations was selected for comparative analyses. The following statistics were used as selection criteria: adjusted coefficient of determination (R^2^), standard error of the absolute estimate (S_yx_) and percentage (S_yx_%)_,_ as well as graphical analysis of residuals.

#### Data mining

In this study we used a data mining technique known as Instance-Based Classification, which uses its own database instances to make estimates of new cases. The technique is based on the premise that certain instances, i.e. whose dimensions form vectors that approximate each other, tend to belong to the same class. This proximity can be measured as the distance between the vectors formed by the independent variables related to the object of study (in the case of this study, sampled trees with dimensions *DBH, H* and *I).*

To calculate the distance between vectors, we used classical Euclidean Distance:

(8) $$d\left(p,q\right)=\sqrt{{\sum_{i=1}^{N}\left(p_{i}-q_{i}\right)}^{2}}$$

Where:

*p*_*i*_ and *q*_*i*_ = measures of any two data points

In this case the formula was applied to DBH, H and I of the sampled trees, namely:

(9) $$d\left(p,q\right)=\sqrt{{\left(\mathrm{DB}H_{p}-\mathrm{DB}H_{q}\right)}^{2}+{\left(H_{p}-H_{q}\right)}^{2}+{\left(I_{p}-I_{q}\right)}^{2}}$$

Where:

*DBH*_*p*_ = diameter of a tree bole *p*

*DBH*_*q*_ = diameter of a tree bole *q*

*H*_*p*_= total height of tree *p*

*H*_*q*_ = total height of tree *q*

*I*_*p*_ = age of tree *p*

*I*_*q*_ = age of tree *q*

The method uses a technique known as *cross validation* where each instance is compared to the others in the sample and the closest (shortest distance) instance is selected from among the rest. In this case the estimated carbon *(C)* of the individual for a given instance will be that value which is least distant.

According to Aha et al. [33] the occurrence of "noise" or instances that not well positioned, is common in this approach. This means that even if the dimensions of a particular instance fall within the range of the others, the value of the dependent variable may have very different values from those of the other instances. This instance is then called "noise" (which is different from an "outlier" or an atypical value and rejected from the data set). In the case of instances in this study, an example of "noise" would be a tree whose values of DBH, H, and Age are similar to other database instances, but whose C value differs significantly from the other instances. Problems then may arise when values of C of these “noise” instances are considered legitimate data values and would lie beyond the normal range, causing errors in the estimate. To minimize the vulnerability of relying on information from instances with these characteristics, a variation in the Instance-Based Classification technique was used. Thus, one can use one nearest neighbor (more susceptible to noise) or create a weighted measure using 3 (or more) nearest neighbors in order to dilute the error. In this case, we use the information from three (or more) nearest neighbors, but favor the closest since it is weighted by the inverse of the distance. This method was recommended in the study by Bradzil et al. [14], in which estimates using between one and five nearest neighbors were made.

The three variations of the method used in this study were:

nearest neighbor:

in this case, *C* was estimated for the tree nearest the one in question;

three nearest neighbors with inverse distance weighting (1/*d):*

in this case, dimensions *DBH* and *H* of the three trees closest to the one in question were used to calculate the inverse Euclidean distance between them as weights in estimating *C;*

three nearest neighbors with inverse distance squared (1/d^2^) weighting:

analogous method to the previous, weighted by the inverse of the squared Euclidean distance.

#### Comparative analyses

To compare the three approaches to estimating the carbon stock of individuals, a paired t test (α = 0.05) was used, comparing the actual value of *C* with its estimates *Ĉ*^*1*^ (regression) *Ĉ*^*2*^ (Volume combined with *BEF* and *R)* and *Ĉ*^*3*^ (data mining).

## Competing interests

The authors declare that they have no competing interests. The views expressed in this publication are those of the authors.

## Authors’ contributions

CRS, J Wojciechowski, A P D Corte, A L Rodrigues, G C B Maas conceived, drafted the manuscript and developed the methodological approaches. All authors read and approved the final manuscript.
